# Transcriptome software results show significant variation among different commercial pipelines

**DOI:** 10.1186/s12864-023-09683-w

**Published:** 2023-11-03

**Authors:** Cung Nawl Thawng, Geoffrey Battle Smith

**Affiliations:** Biology Department and Molecular Biology Program, New Mexico State University, Las Cruces, NM USA

**Keywords:** Transcriptome software, RNA-Seq, Low radiation, Pipeline, Fold-changes, Model organisms

## Abstract

**Background:**

We have been documenting the biological responses to low levels of radiation (natural background) and very low level radiation (below background), and thus these studies are testing mild external stimuli to which we would expect relatively mild biological responses. We recently published a transcriptome software comparison study based on RNA-Seqs from a below background radiation treatment of two model organisms, *E. coli* and *C. elegans* (Thawng and Smith, BMC Genomics 23:452, 2022). We reported DNAstar-D (Deseq2 in the DNAstar software pipeline) to be the more conservative, realistic tool for differential gene expression compared to other transcriptome software packages (CLC, Partek and DNAstar-E (using edgeR). Here we report two follow-up studies (one with a new model organism, *Aedes aegypti* and another software package (Azenta) on transcriptome responses from varying dose rates using three different sources of natural radiation.

**Results:**

When *E. coli* was exposed to varying levels of K40, we again found that the DNAstar-D pipeline yielded a more conservative number of DEGs and a lower fold-difference than the CLC pipeline and DNAstar-E run in parallel. After a 30 read minimum cutoff criterion was applied to the data, the number of significant DEGs ranged from 0 to 81 with DNAstar-D, while the number of significant DEGs ranged from 4 to 117 and 14 to 139 using DNAstar-E and the CLC pipelines, respectively. In terms of the extent of expression, the highest foldchange DEG was observed in DNAstar-E with 19.7-fold followed by 12.5-fold in CLC and 4.3-fold in DNAstar-D. In a recently completed study with *Ae. Aegypti* and using another software package (Azenta), we analyzed the RNA-Seq response to similar sources of low-level radiation and again found the DNAstar-D pipeline to give the more conservative number and fold-expression of DEGs compared to other softwares. The number of significant DEGs ranged 31–221 in Azenta and 31 to 237 in CLC, 19–252 in DNAstar-E and 0–67 in DNAStar-D. The highest fold-change of DEGs were found in CLC (1,350.9-fold), with DNAstar-E (5.9 -fold) and Azenta (5.5-fold) intermediate, and the lowest levels of expression (4-fold) found in DNAstar-D.

**Conclusions:**

This study once again highlights the importance of choosing appropriate software for transcriptome analysis. Using three different biological models (bacteria, nematode and mosquito) in four different studies testing very low levels of radiation (Van Voorhies et al., Front Public Health 8:581796, 2020; Thawng and Smith, BMC Genomics 23:452, 2022; current study), the CLC software package resulted in what appears to be an exaggerated gene expression response in terms of numbers of DEGs and extent of expression. Setting a 30-read cutoff diminishes this exaggerated response in most of the software tested. We have further affirmed that DNAstar-Deseq2 gives a more conservative transcriptome expression pattern which appears more suitable for studies expecting subtle gene expression patterns.

**Supplementary Information:**

The online version contains supplementary material available at 10.1186/s12864-023-09683-w.

## Background

The RNA sequencing (RNA-seq) approach has been a popular method of choice for many researchers who want to study gene expression in the biological fields [1–3]. As the method’s level of resolution for gene expression has increased, the cost of RNA-seq has lowered, giving researchers unique insights into biological behavior. Concurrently, many RNA-seq analysis tools/software have been developed in recent years to complement and expand researchers’ abilities to analyze the resultant data [4–6]. Basically, the RNA-seq analysis steps include 1) quality control analysis of the raw RNA-seq by trimming and removing low quality sequences, 2) mapping or alignment of RNA-seq into the database if genome information is available or de novo mapping if genome information is not available, 3) quantification and normalization of the transcript, and 4) differential gene expression analysis [7].

Many RNA-seq analysis software are developed to encompass these steps and each step in the analysis pipeline has different parameters that can be adjusted based on the software/tools [4, 8–10]. It is possible that one parameter of RNA-seq pipeline is adjustable in one software which is invariable with another software. Commercial transcriptome analysis software like CLC, Partek and DNAstar have all steps of analysis in one package. For example, CLC has its own mapping system while normalization and differential gene expression is done by algorithms similar to edgeR [11]. Likewise, DNAstar has SeqManNGen for mapping, while edgeR and DESeq2 are optionally available for normalization and differential gene expression. So, in addition to the importance in designing an experiment and choosing the number of biological replicates, but also the appropriate software to use and what parameters to set up in each analysis step are also important factors for the success of RNA-seq experiments [12–16].

Several comparative studies on differential expression software for transcriptome analysis have been reported and DESeq2 is a commonly used tool [17–21]. In a recent study, we compared four commercially available transcriptome software for differential gene expression studies using RNA-seq data from *E. coli* and *C. elegans* treated with different source of radiation, and we found that DESeq2 (in the DNAstar package) was a more conservative and appropriate software for treatments expected to give subtle gene expression patterns [22]. In this follow-up study, we evaluated another set of RNA-seq data from *E. coli* exposed to different radiation doses of ^40^K using CLC, DNAstar-E (edgeR) and DNAstar-D (DESeq2) for differential gene expression. We also evaluated RNA-seq data sets from the *Aedes aegytpi* mosquito treated with different sources of radiation using DNAstar-D (DESeq2), DNAstar-E (edgeR), CLC and Azenta (DESeq2).

## Results

### Transcriptome software/pipeline comparison

**Fig. 1:**
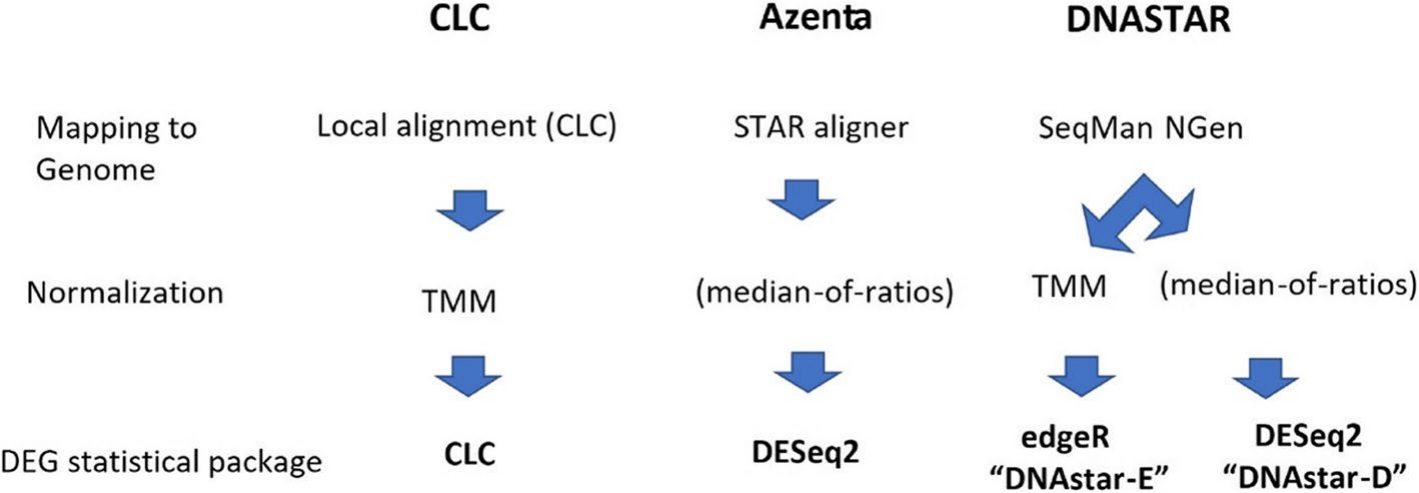
The different program mapping, normalization and statistical approaches used for each of the software pipelines used in this study

Figure 1 compares a new software package (Azenta) with two of the previously used softwares (CLC and DNASTAR) for differential gene expression analysis of RNA-seq in a new model organism, *Ae. aegypti*. In a new *E. coli* RNA-seq analysis comparing different levels of a natural radiation source (KCl), CLC and DNASTAR softwares were used. Important software variables are as follows: The CLC package has two options available for mapping, namely local alignment and global alignment, whereas Trimmed Mean of M-values (TMM) is used in normalization. The differential gene expression statistical package in CLC is based on General Linear Model with a negative binominal distribution algorithm (https://digitalinsights.qiagen.com). Azenta uses STAR aligner for mapping RNA-seq to a reference genome and differential gene expression is analyzed by DESeq2 with normalization by median-of-ratios (https://www.azenta.com). In DNASTAR, SeqMan NGen is integrated in the program for mapping to reference genomes while BioConductor’s DESeq2 and edgeR are optionally available for differential gene expression calculation with its own normalization method (https://www.dnastar.com/workflows/rna-seq/). As shown in Fig. 1, the three softwares use different mapping systems, using either TMM or median of ratio for Normalization. For DEG analysis, the DESeq2 statistical package is shared between DNAStar-D and Azenta, whereas DNAStarE uses edgeR and CLC uses its own statistical package (Fig. 1).

### Differential gene expression of *E. coli*

**Fig. 2:**
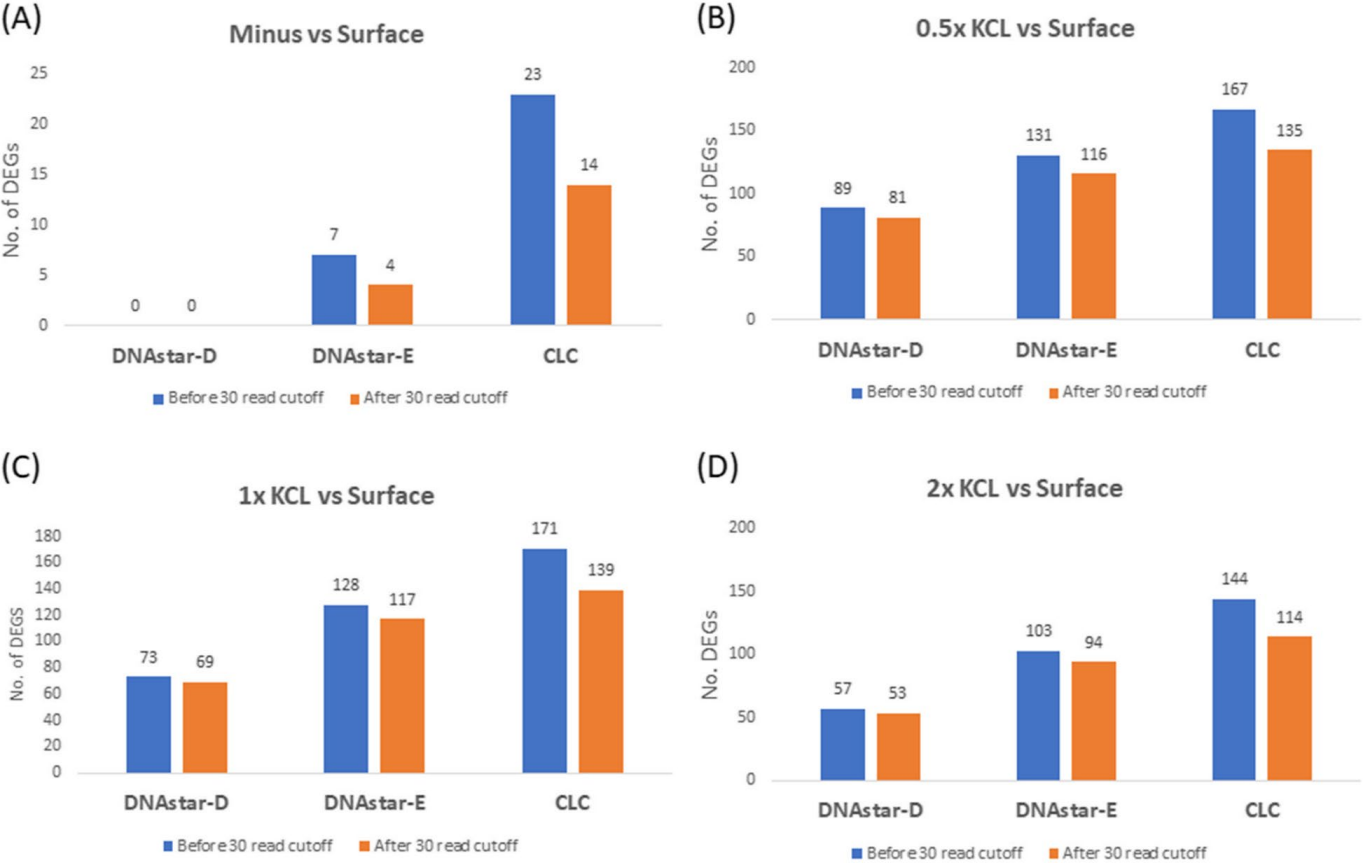
Number of differentially expressed genes (DEGs) in the *E. coli* study with and without imposing a 30-read cutoff criterion (Data includes only data with greater than 30 reads in all replicates treatments). Cells were exposed to 4 treatments underground at WIPP: Minus = Minus radiation from cells grown in a steel vault, KCL = Cells grown in a KCL-Irradiator (0.5 × KCL = 7.04 kg (31.8 nGy/hr), (1 × KCL = 14.02 kg (81.4 nGy/hr) and (2 × KCL = 22.55 kg (152.9 nGy/hr), Surface = Cells grown at surface as a control (natural background radiation)

**Fig. 3:**
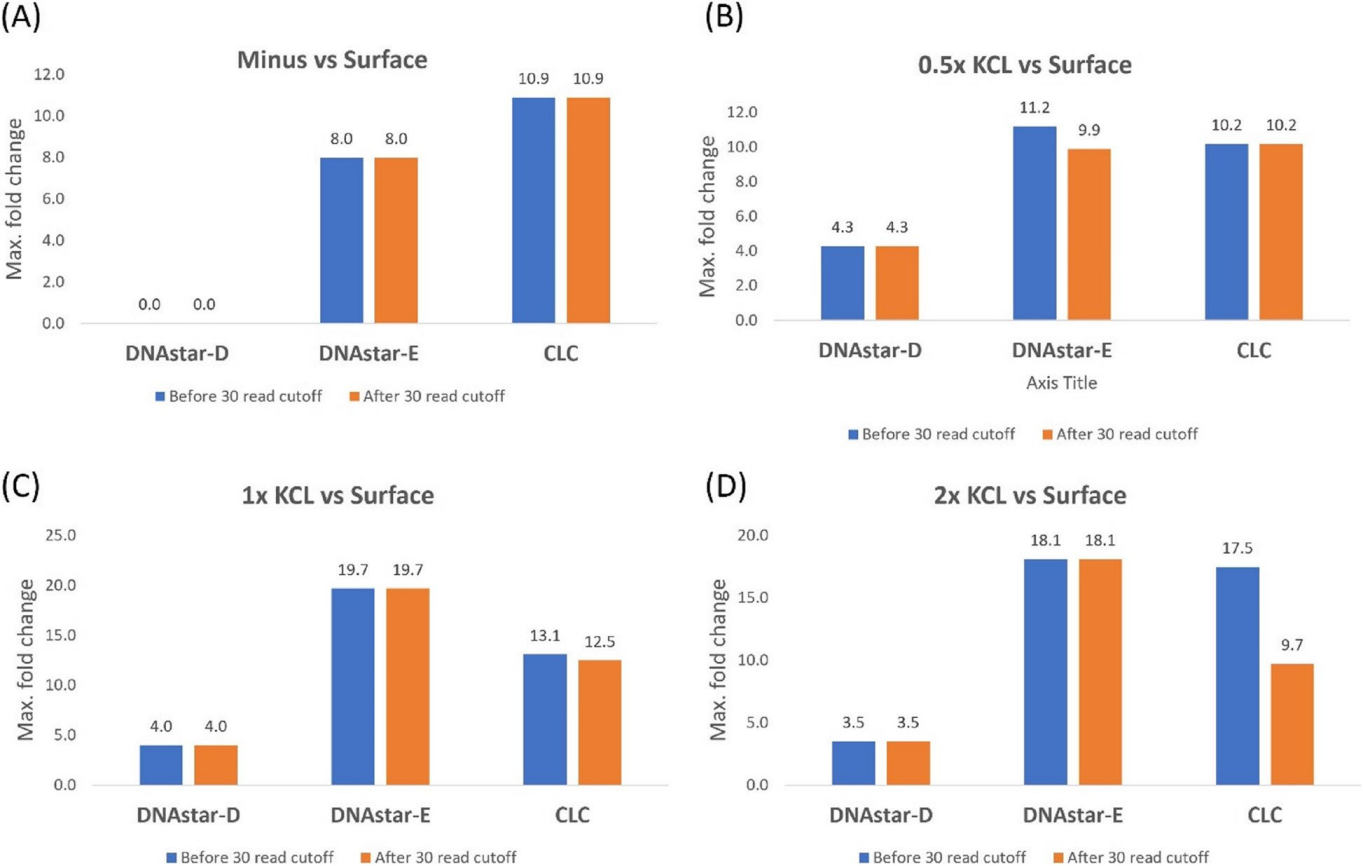
Maximum fold change observed in DEGs of *E. coli* with and without the 30-read cutoff

Differentially expressed genes (DEGs) were analyzed using CLC and DNASTAR software based on *E. coli* being treated with different doses of ^40^K underground compared to a control dose at the NMSU surface. In a previous study, when we used a minimum raw read cutoff of 30, this removed exaggerated (eg. 25 – 180) fold-change effects with these types of low-level radiation treatments. Here we tested that criterion and the raw data of DEGs were analyzed with and without a 30 read cutoff (Fig. 2). Using the NMSU surface as a control where the cells were exposed to natural surface levels of radiation, we again documented that imposing a 30-read minimum cutoff lowered the number of DEGs, but with this dataset, the differences were not as large as we’ve documented in other studies cited above. The main effect is that, as we’ve documented previously, the different softwares gave quite different numbers of DEGs, with CLC yielding the most (14–139), DNAStar-D the least (0–81) with DNAStar E being intermediate (4–117) (Fig. 2, with the 30 read cutoff). Quantifying the extent of expression by examining maximum fold change with the 30 minimum cutoff (Fig. 3), CLC ranged from 9.7 – 12.5, DNAStar- E (8.0–19.7) and DNAStar-D yielded the lowest fold-change (0—4.3). This is the same pattern that we’ve seen in previous studies using different radiation treatments with *E. coli* and the *C. elegans* nematode [21]. It is obvious that different softwares gave different numbers of DEGs and different extents of expression, but what is most interesting is that the three software packages give the same pattern of results, with DESeq2 (DNAStart-D) being consistently the most conservative.

### Differential gene expression of mosquito (*Ae. aegypti*)

**Fig. 4:**
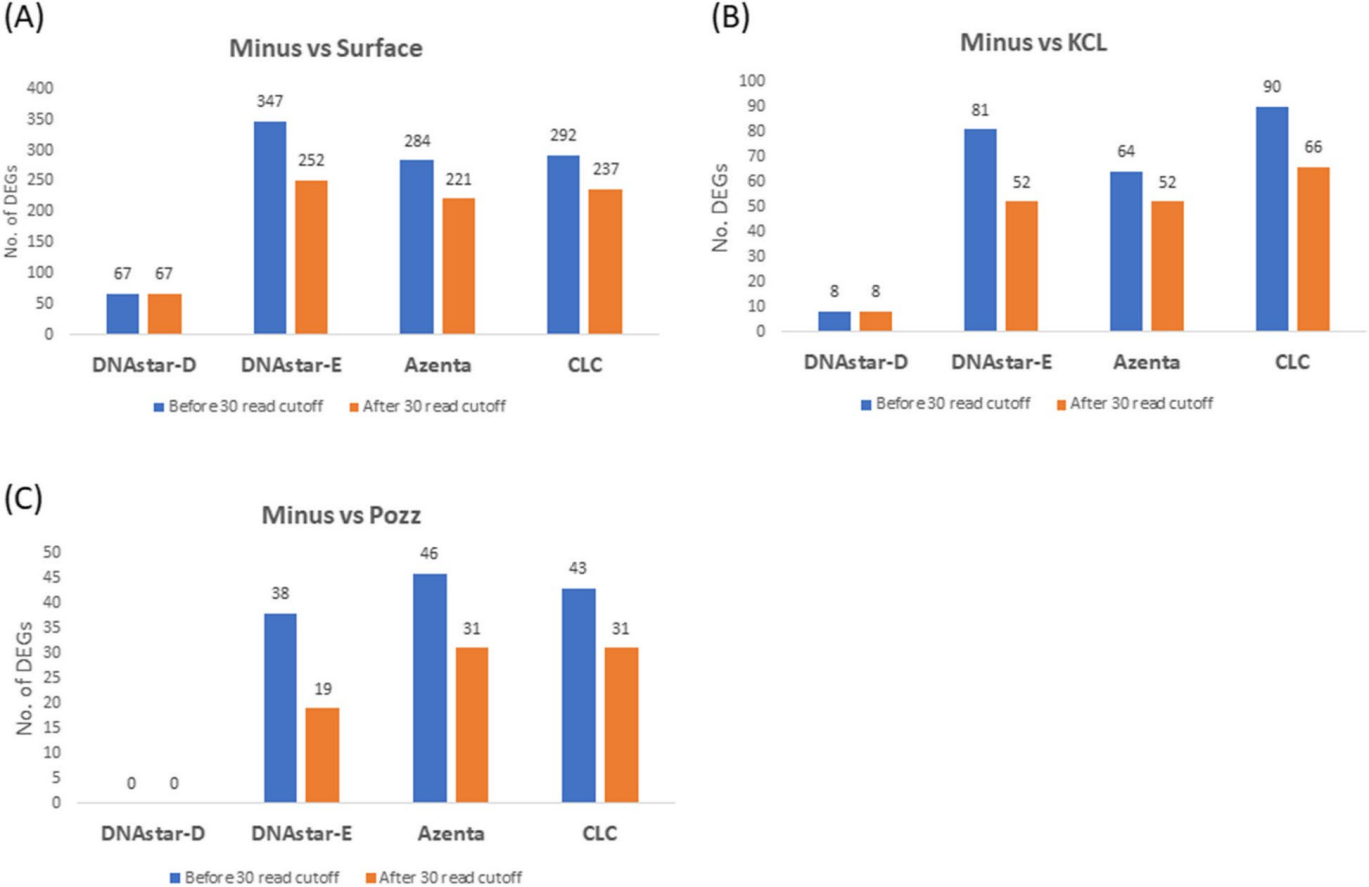
Number of differentially expressed genes (DEGs) in mosquito with and without imposing a 30-read cutoff criterion (Data includes only data with greater than 30 reads in all replicates treatments). Mosquito were exposed to 3 treatments underground at WIPP: Minus = Minus radiation from mosquito grown in a steel vault (0.004 nGy/hr), KCL = Mosquito grown in a KCL-Irradiator (81.4 nGy/hr), Pozz = Mosquito grown in Pozzolan-Irradiator (70.7 nGy/hr). Surface = Mosquito grown at surface as a control (natural background radiation) (35 mGy/hr)

**Fig. 5:**
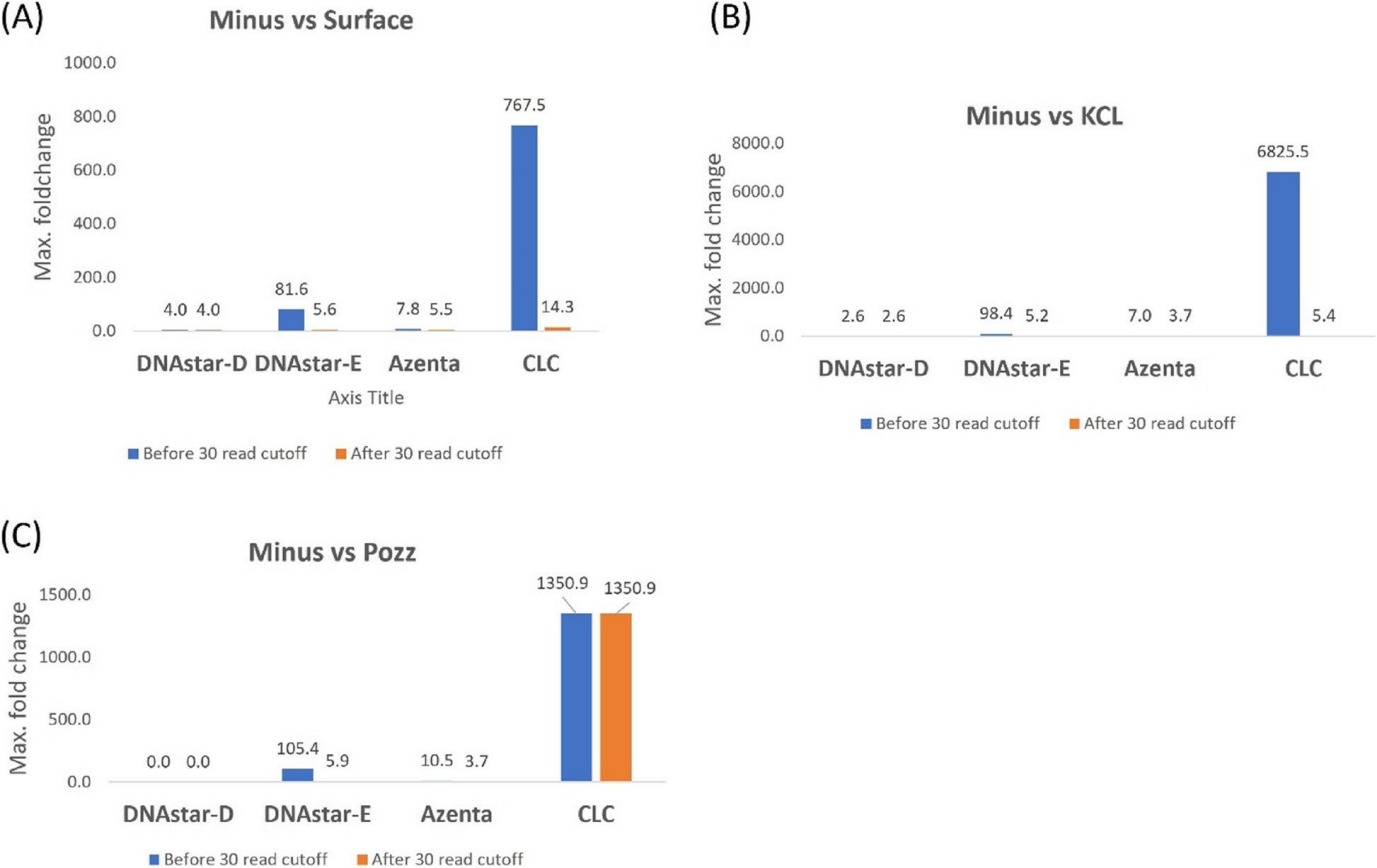
Maximum fold change in DEGs of mosquito

The transcriptome of mosquito was first analyzed by the Azenta software package (the company which performed RNA sequencing), and this analysis was compared to our analyses using DNAStar (using DESeq2 or edge R statistics) and CLC. Each analysis produced lists of differentially expressed genes (DEGs) from the minus group (abnormally low radiation treatment) versus different sources of natural radiation amendments, namely incubators supplemented with KCl or pozzolan, in comparison with control mosquitos incubated at the WIPP surface. In agreement with our previous study [22], a 30-read cutoff criterion again lowered the number of DEGs in all comparisons except in the case of DNAStar-D, which gave the lowest DEGs and was unaffected by the cutoff (Fig. 4). The CLC and DNAstar-E pipelines showed the highest number of DEGs, with Azenta intermediate and DNAStar-D giving the lowest numbers. When the highest fold-change of DEGs was analyzed without the 30-read cutoff, the most exaggerated fold change was observed in CLC with up to 6825-fold, DNAstar-E 105-fold, 10-fold in Azenta and 4-fold in DNAstar-D (Fig. 5). After the 30 read cutoff was applied, most of these exaggerated fold changes were removed except in CLC analysis where 1351 fold change still remained the highest (Fig. 5). Again, the 30 read cutoff had no effect on the maximum fold change in DNAstar-D.

Repeated analysis supported our previous findings in *E. coli* – that the DNAStar package provided more conservative DEG calculations that are likely more appropriate for these studies that are testing a relatively subtle radiation effect. For example, comparing the total number of DEGs among the three treatments (75 for DNAStar-D and 304 for Azenta, Fig. 4), the Azenta analysis resulted in more than four times as many DEGs as the DNAStar-D package yielded. Both Azenta and DNAStar-D used DESeq2 for analyzing differential gene expression, therefore the difference in number of DEGs apparently is the result of the mapping system difference between the two softwares. In Figs. 2, 3, 4 and 5, four treatment comparisons were carried out using *E. coli* and 3 comparisons were used for *Ae. aegypti*. Examining both the number of DEGs and the extent of expression (as measured by fold-change) resulted in 14 data sets. Of the 14 comparisons using the 30 read cutoff criterion, the CLC software yielded the highest value in 12 of the 14 comparisons.

## Discussion

We have been studying the cell and organismal response to the deprivation of normal background levels of radiation by incubating different model organisms in a salt deposit 658 m underground at the Waste Isolation Pilot Plant (WIPP) outside of Carlsbad, NM (example publications [22–26]). The WIPP is the United States only deep repository for weapons-related nuclear waste, and, since the 1’st waste emplacement in 1999, 71,000 metric tons of transuranic waste have been reposited. Previously this nuclear waste had been stored aboveground at 34 facilities, and, as a result of waste emplacement at WIPP, these temporary storage sites have been reduced to 19 (chrome-extension://efaidnbmnnnibpcajpcglclefindmkaj/https://wipp.energy.gov/Library/TRUwaste/ATWIR-2021_CBFO_Final.pdf).

**Fig. 6:**
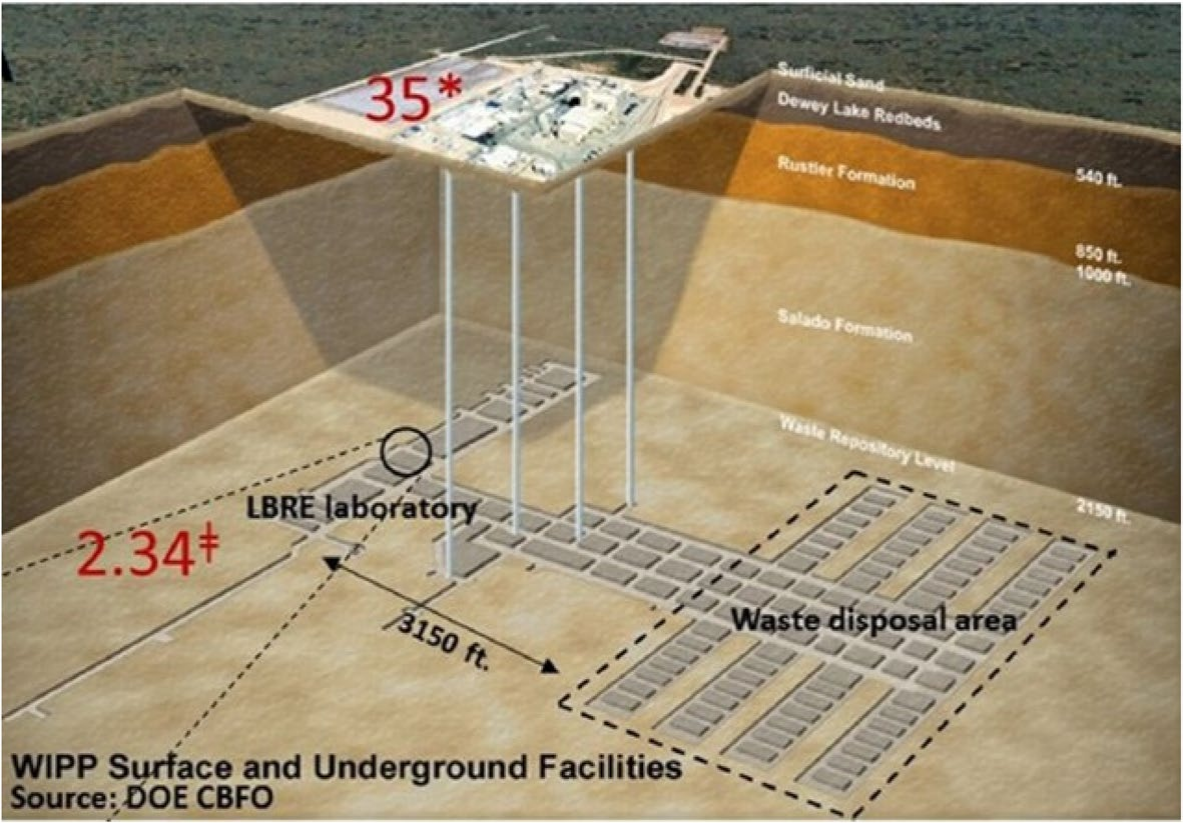
Location of the Low Background Radiation Experiment (LBRE) lab 2150 ft. below the surface and 3150 ft. from the nuclear waste at the Waste Isolation Pilot Plant (WIPP). The numbers in red indicate the radiation dose rates at the surface (35 nGy/hr) and in the LBRE lab (2.34 nGy/hr). Note, the pre- WWII steel vault adjoins the LBRE lab and has a lower radiation dose rate (0.004—0.9 nGy/hr, depending on the isotopes in the growth media). Adapted from Castillo and Smith, 2017 [27]

At the north end of the mine, one km from the nuclear waste there is a large experimental area that has been used by physicists (eg. the Enriched Xenon Observatory experiment, https://www-project.slac.stanford.edu/exo/default.htm) and biologists (see eg. publications above) to perform measurements and experiments in a very low background, radiologically quiet environment. It is of course ironic to be performing radiologically sensitive measurements in a nuclear waste repository, but it does give credence to isolating the waste in a halite deposit where one km of salt is more than enough to completely shield the radiation. A depiction of the WIPP repository and the location of our Low Background Radiation Experimental (LBRE) lab is shown in Fig. 6.

The transcriptome experiments we have carried out are a basic comparison to the radiation-reduced treatment from cells incubated underground in a pre-WW II steel vault (to further shield cells from radiation) and this is compared to a control aboveground that is experiencing background radiation. Over the years we have developed additional controls where underground we add back a natural source of radiation, for example KCl (which has 0.01% ^40^K) or we use a volcanic source like Pozzolan to mimic background. In our experiments, we are testing treatments and controls that differ by relatively little radiation, the difference between background radiation (which is low) and below-background (which is very low).

In our recent work, we’ve documented surprisingly high fold-changes in many of the differentially regulated genes. For example, using 3 different commercial transcriptome analyses programs (CLC Genomic, Partek and DNAStar-RPKM), 24 of the 26 major sperm related *C. elegans* genes were more than 10-fold up-regulated (range 10.5 – 42.9, Van Voorhies et al. 2020 Table 1 [26]) in the absence of normal levels of radiation. Considering the low vs lower radiation treatment differences, we were surprised of the extent of up-regulation, but since all three pipelines showed this, the manuscript was published. Recently, we’ve taken a more careful look at the different statistical analyses options and, adjusting some of the parameters of the programs, found that imposing a less than 30 read cutoff lowered the maximum-fold change for all the *C. elegans* DEGs from 936.5 to 14.6 (CLC Genomics) and from 761.3 to 20.8 (DNAStar using EdgeR). Interestingly, for the DNAStar option using the DESeq2 statistical package, the 30 read-cutoff did not make any difference, with both options showing an already-conservative 3.3 maximum fold-change (Fig. 3B in Thawng and Smith, 2022 [22]). The more conservative number and extent of regulation of the *C. elegans* genes reported in 2022 did not change the biological interpretation we had reached in our 2020 VanVoorhies paper, but the more refined use of these commercial softwares gave more realistic estimates of the extent of differential expression.

**Table 1:** Different source of radiation treatment for mosquito culture. The surface value were adapted from Chiou & Hayes, 2004 [28]

Treatment	Dose Rate (nGy/hr)
Minus (WIPP)	0.004
KCl	81.4
Pozzolana (*n* = 2)	70.7
Surface (Chiou & Hayes, 2004) [28]	35

Without the short read cut-off criterion, both the number of DEGs and the extent of fold-changes are typically exaggerated. It is also important to examine the raw reads and normalization data of the significant genes because exaggerated fold-changes in some of the genes may still persist because of mapping. Finally, the extent of the fold-change cut-off (eg., 2-fold or 1.5-fold in this case) is important to consider when comparing two or more softwares for transcriptome analysis since the percentage of agreement between softwares will be increased as the fold-change cutoff decreases. This is especially true when a very conservative transcriptome software like DNAstar-D is used for comparison with others (Fig. 2 in Thawng and Smith, BMC, 2022 [22]).

As mentioned in the introduction, another consideration should be given to each software mapping parameters. For example, in CLC, mapping parameters include mismatch cost, insertion cost, deletion cost, length fraction, similarity fraction, local or global alignment while in DNAstar, SeqMan NGen mapping parameters including Mer size, minimum match %, maximum aligned length and maximum gap size which are optionally available. Adjusting and optimizing those parameters could also affect the mapping results of each significant genes. In our studies, in order to make valid comparisons, we used the default parameters of each software analyses of transcriptome data.

In looking at the raw mapping data of the specific gene with the most exaggerated fold change (6825-fold), it was apparent that the total gene reads were the combination of both intron and exon reads. Therefore, when using CLC, we would recommend 30 read cutoff should be applied to total exons reads not total gene reads when analyzing biological models such as mosquito which of course have introns.

Our previous data are consistent with the current results in which the imposition of a 30-read cutoff eliminated most of the spuriously high DEGs and fold-changes. Consistent with our current and recent [22] studies, DESeq2 performed better than other methods in terms of FDR control. Other researchers have shown the DESeq-2 retains statistical power and stability across sample sizes [6, 9, 29, 30]. In this study, we also demonstrated that DESeq2 performed better in terms of being more consistent and conservative among the tested methods. Of course, there are other pipeline steps like what mapping program is used that also are important in determining outcomes. For example, both the Azenta and DNAStar-D packages use DESeq2 statistical analysis (and the same median of ratios normalization step), yet the total numbers of *Ae. aegypti* DEGs among 3 treatment comparisons differed by 4-fold. The use of different mapping programs (Star aligner for Azenta and SeqMan NGen for DNAStar), most likely accounts for this difference.

In our radiation transcriptome studies to date in which we’ve used more than one software package, the CLC Genomics has generally resulted in the highest numbers of DEGs and fold-changes [22, 26]. The current study demonstrates the same pattern: in Figs. 2, 3, 4 and 5, four treatment comparisons were carried out using E. coli and 3 comparisons were used for Ae. aegypti. Examining both the number of DEGs and the extent of expression as measured by fold-change resulted in 14 data sets. Of the 14 comparisons using the 30 read cutoff criterion, CLC software yielded the highest value in 12 of the 14 comparisons. When shared DEGs were analyzed among the software, it varies widely based on the organisms.

When we analyzed the percentage agreement among the softwares, in the *E coli* study, percent agreement ranged from 0 to 53% (based on CLC as it showed the highest number of DEGs) among different software (Supplementary Figure 1). In the *Ae. aegypti* study, it ranged from 0 to 25% agreement among all the tested software (Supplementary Figure 2). In this study, we focused on the DNAstar-D software because we think it is, due to its more conservative results, the most appropriate software for studies expected to give subtle gene expression responses. As the DNAstar-D is more conservative in terms of resulting in lower number of DEGs and lower fold-change values compared to other softwares, the agreement percentage is also low compared to the other softwares. For example, in the *Ae. aegypti* mosquito data, the four softwares (DNAstar-D, DNAstar-E, Azenta and CLC) agree on only about 25% of the number of DEGs. However, if we remove DNAstar-D, the agreement among the other three (Azenta, CLC and DNAstar-E) would be more than 75%. However, this does not necessarily mean one should choose CLC and Azenta because of the high agreement between them. Instead, we would suggest focusing on the software that is appropriate to the experiment (in this example, one which yields the relatively small treatment differences one would expect from experiments looking for subtle responses).

On the other hand, the percentage agreement can be increased by changing the fold-change cutoff value from 2-fold to 1.5-fold. For instance, if the fold change cutoff was set for CLC and DNAstar-D at 1.5-fold, the number of significant DEGs for DNAstar would increase from 67 to 418 and the number of significant DEGs for CLC would increase from 292 to 771. Therefore, the number of DEGs that are different between DNAstar D and CLC would decrease from 4.3 times (67 to 292) to 1.8 times (418 to 771). Subsequently, the number of DEGs in agreement would increase to 47.6%.

The yearly cost of these commercial pipelines is not insignificant, ranging from 2000–3200 USD per year per software package. So, it is an interesting and important decision for lab directors to balance, for example, the employee time spent in learning to write code and implement “in-house” analyses vs. saving the labor and time costs and purchasing standard commercial packages that are likely to be more lab-to-lab comparable. In our lab, we have chosen to prioritize employee time in experimental design and biological science instead of students writing code, for example. Another consideration is the running time for each software which is of course dependent on the genome size. *E. coli* data analyses run faster than mosquito analyses, and so it takes 24 h (prokaryote) to 48 h (eukaryaote) for turn-around times. In our lab, the computer that we used has 256 GB RAM which is the key parameter to ensure expedited processing for RNAseq analyses of large genomes.

## Conclusions

RNA sequencing is a powerful method to study gene expression and is useful in identifying important, and possibly novel genes in biological studies [1]. However, the complexity of RNA-seq analysis software methods may limit its applications and experimental interpretations [10]. In this study, we have shown that, from the same RNA sequence raw data, different softwares resulted in quite varied results. Using three different biological models (bacteria, nematode and mosquito) in four different studies testing very low levels of radiation (Van Voorhies et al. [26]; Thawng and Smith, [22]; current study), the CLC software package resulted in what appears to be an exaggerated gene expression response in terms of numbers of DEGs and extent of expression. On the other hand, using the DESeq-2 statistical analysis package option in the DNAStar software consistently gave the most conservative estimates of the number and extent of differentially expressed genes [22, 26]. We believe these four studies represent experiments that are testing subtle, but important environmental stimuli, and thus we hope these results and suggestions will help other researchers who are testing variables expected to give small but biologically significant effects. It is important for researchers who are not experts in bioinformatic programming (like us!) to be careful in choosing which transcriptome software and parameters to use for their RNA studies. We therefore recommend for transcriptome studies utilizing commercial software for analysis: 1. Choose the appropriate software depending on the experimental design and expected results. 2. If possible, compare results using more than one software package. 3. Apply a 30-read cutoff on raw data of significant genes. 4. Test other fold-change cutoffs than the standard 2.0 cutoff to test the effect of this criterion on how different software results agree. 5. Carefully consider the programs’ adjustable parameters in order to optimize the program to the data set being used.

## Methods

### *E. coli* culture conditions

**Fig. 7:**
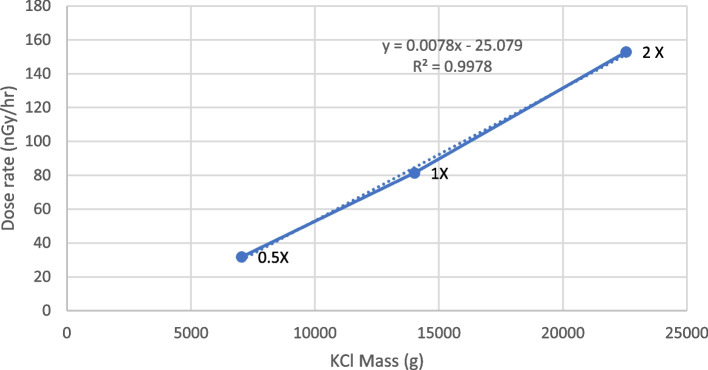
KCl Irradiator dose response

A minus 80 °C frozen glycerol stock of *Escherichia coli* K-12 (ATCC 10798) was struck on TGY agar plates and incubated at 30 °C for 1 day. Four separate colonies from the agar plate were inoculated into four TGY broths (2 mL) in 15 mL tubes and incubated at 30 °C 250 rpm for 2 days. Then 20 µL of cultures were transferred to fresh TGY broth media (2 mL) and incubated overnight at 30 °C 250 rpm. After 16–18 h of incubation, the cultures were transported to WIPP and two of the biological replicates were refrigerated in a Surface lab until use the next day, and two of the reps were diluted 25 µL /10 mL of fresh TGY. The diluted cultures (1.5 mL) were transferred into the top 6 wells of four 24-well plates (MIDSCI, St. Louis, MO) and incubated at 30 °C 250 rpm. The cells incubated 24 h underground at WIPP which represented a pre-incubation before the cells were transferred again to initiate a 3.5-h incubation. Plate counts and optical densities were measured at time-zero and after 3.5 h and cells were harvested for RNA (see below). The process was repeated with the other two biological replicates of cells which had been refrigerated for 24 h. In this way, four biological replicates were carried out in this experiment as described previously [22]. The 24-well plates were incubated underground at WIPP in four Peltier incubators (Sheldon Lab model SR13P) under the following conditions: 1. In a 15.2 cm-thick vault made from pre-World War II steel (our “minus” treatment), and in three incubators with three different amounts of KCl (a natural source of radiation having 0.01% ^40^K) ranging from 7 – 22.6 kg. A linear relationship was observed between mass of KCl and dose rate in the three incubators (Fig. 7). For surface control experiments (normal/natural background radiation), the cultures were conducted in a similar manner as above except incubation of the cultures were performed at New Mexico State University (NMSU).

### RNA collection and sequencing (*E. coli*)

RNA samples were prepared for bulk RNA-sequencing as previously described [22]. Briefly, on the 2’nd day of incubation underground, exponential phase (3.5 h) cultures of *E. coli* was harvested as follows: 1 mL of RNA protect solution was added into 0.5 mL of culture and kept at room temperature for 5 min after mixing well. Cell pellets were harvested by centrifugation at 12,000 rpm for 5 min. The supernatant was decanted, and the pellet was kept at -20 °C. RNA was extracted from the cell pellet using an RNA isolation kit (RNeasy@ Mini Kit, QIAGEN) according to manufacturer’s instruction. The quantity and quality of RNA was evaluated by Nano drop and by running on agarose gel electrophoresis. The total RNA samples were sent for sequencing at Novogene (Sacramento, CA). For *E. coli* library construction, rRNA was removed using the Ribo-Zero kit that leaves mRNA. First, mRNA was fragmented randomly by adding fragmentation buffer, then the cDNA was synthesized by using mRNA template and random hexamers primer, after which a custom second-strand synthesis buffer (Illumina), dNTPs (dUTP, dATP, dGTP and dCTP), RNase H and DNA polymerase I were added to initiate the second-strand synthesis. This was followed by purification by AMPure XP beads, terminal repair, polyadenylation (for bacteria), sequencing adapter ligation, size selection and degradation of second-strand U-Contained cDNA by the USER enzyme. The strand-specific cDNA library was generated after the final PCR enrichment. Library concentration was first quantified using a Qubit 2.0 fluorometer (Life Technologies), and then diluted to 1 ug/µl before checking insert size on an Agilent 2100 and quantifying to greater accuracy by quantitative PCR (Q-PCR) (Library activity > 2 nM. Qualified libraries were sequenced on an Illumina Nova Seq 6000 Platform (Illumina, San Diego, CA, USA) using a paired-end 150 run (2 × 150 bases). The raw reads RNA-seq data were deposited at NCBI accession no. PRJNA910508. Summary of RNA-seq quality can be found at Supplementary Table 1.

### Data analysis

For the transcriptome analyses, CLC Genomic Workbench 12.2 (Qiagen Bioinformatics, Germantown, MD, USA) was used. All RNA Seq data were screened for False Discovery Rate (FDR) and were accepted if FDR < 0.05 and 2-fold change. Raw RNA sequences were trimmed, aligned, and mapped against the reference genome of *E. coli* K-12 MG1655 (NC_000913.3) in CLC program with the following parameters: 2 maximum mismatches, 90% minimum similarity fraction, and 10 maximum hits per read for mapping. The raw RNA-seq were also analyzed by DNAStar (Madison, Wisconsin, USA) with SeqMan N Gen (version 17.2.1.61) for mapping. Differential gene expression was analyzed by DNAstar (DESeq2) and DNAstar (edgeR). Other than using a 30-base read cutoff as indicated, default parameters of each software tool was used for all RNA-seq analyses. For 30 read cutoff criteria, we used only data that had greater than 30 reads in all eight replicates (4 replicates from control and 4 replicates from treatment) [22].

### Mosquito rearing and radiation treatment

Male *Ae. aegypti* were subjected to radiation treatments at WIPP designed to test different radiation sources to test the effect of the “quality” of radiation at similar quantities (Table 1). The sources used for the radiation quality study were 1. Natural background (incubated in our surface lab at WIPP), 2. Below background at WIPP (underground in the steel vault, the “minus” treatment), 3. pozzolana supplemented treatment, and 4. KCl supplemented treatment (Table 1). Mosquitoes were incubated between 23.5 and 24.5 °C and provided with 20% sucrose. Ae. aegypti were observed over a 16-day span to assess mortality, environmental conditions (temperature and humidity), and at the conclusion of the study, they were sampled for transcriptome analysis in triplicate.

### RNA preparation and sequencing (mosquito)

RNA extractions were performed using a Qiagen RNeasy Mini Kit according to manufacturer suggested protocol with minor modification. Throughout, reagents and samples were kept cool on ice. Quality of RNA samples was evaluated using gel electrophoresis to observe bands, Nanodrop absorbance ratios (A260/280), and with RIN [31]. Extracted RNA were sent to Azenta Life Science for RNA sequencing and bioinformatics analysis. The raw RNA-seq were deposited at NCBI accession no. PRJNA915031. Summary of RNA-seq quality can be found at Supplementary Table 2.

### Bioinformatic analysis

RNA-seq raw reads were trimmed to remove possible adapter squences and nucleotides with poor quality using Trimmomatic v.0.36. The trimmed reads were mapped to the Aedes aegypti LVP AGWG reference genome available on ENSEMBL using the STAR aligner v.2.5.2b. The STAR alinger is a splice aligner that detects splice junctions and incorporates them to help align the entire read sequences. Unique gene hit counts were calculated by using featureCounts from the Subread package v.1.5.2. The hit counts were summarized and reported using the gene_id feature in the annotation file. Only unique reads that fell within exon regions were counted. After extraction of the gene hit counts, the gene hit counts table was used for downstream differential expression analysis. Using DESeq2, a comparison of gene expression between customer-defined groups of samples was performed. The Wald test was used to generate *p*-values and log2 fold changes. Genes with an adjusted *p*-value < 0.05 and absolute log2 fold change > 1 were called as significant differentially expressed genes for each comparison. The trimmed RNA-seq were also analyzed by DNASTAR. Seq Man Ngen was used for mapping to the genome databased and DNAstar (DESEq2) was used for normalization and differential gene expression. For 30 read cutoff criteria, we used only data that had greater than 30 reads in all six replicates (3 replicates from control and 3 replicates from treatment) [22].

### Supplementary Information

**Additional file 1: Supplementary Figure 1.** Shared DEGS of E. coli among different software. (A) Minus vs Surface treatment, (B) 0.5x KCL vs Surface treatment, (C) 1x KCL vs Surface treatment and (D) 2x KCL vs Surface treatment. **Supplementary Figure 2.** Shared DEGs of mosquito (Aedes aegypti) among different software. (A) Minus vs Pozzalan treatment, (B) Minus vs Surface treatment and (C) Minus vs KCL treatment.** Supplementary Table 1. **The raw reads obtained from RNA-seq of *E. coli* in this study.** Supplementary Table 2.** The raw reads obtained from RNA-seq of Ae. Aegypti.

## Data Availability

All data generated or analyzed during this study are included in this published article. The raw RNA sequences obtained in this study were deposited at NCBI database (Accession number PRJNA910508 and PRJNA915031).

## Abbreviations

WIPP: Waste Isolation Pilot Plant
DEG: Differentially expressed genes
NGS: Next-Generation Sequencing
