# Supplementary material for: Transcriptome software results show significant variation among different commercial pipelines
Source: BMC Genomics. 2023 Nov 3;24:662. doi: 10.1186/s12864-023-09683-w (PMC10623858; doi:10.1186/s12864-023-09683-w)
Supplement: Supplementary file 1 — Additional file 1: Supplementary Figure 1. Shared DEGS of E. coli among different software. (A) Minus vs Surface treatment, (B) 0.5x KCL vs Surface treatment, (C) 1x KCL vs Surface treatment and (D) 2x KCL vs Surface treatment. Supplementary Figure 2. Shared DEGs of mosquito (Aedes aegypti) among different software. (A) Minus vs Pozzalan treatment, (B) Minus vs Surface treatment and (C) Minus vs KCL treatment. Supplementary Table 1. The raw reads obtained from RNA-seq of E. coli in this study. Supplementary Table 2. The raw reads obtained from RNA-seq of Ae. Aegypti. [file 12864_2023_9683_MOESM1_ESM.docx]

**Supplementary data**

**Supplementary Figure1.** Shared DEGS of E. coli among different software. (A) Minus vs Surface treatment, (B) 0.5x KCL vs Surface treatment, (C) 1x KCL vs Surface treatment and (D) 2x KCL vs Surface treatment.


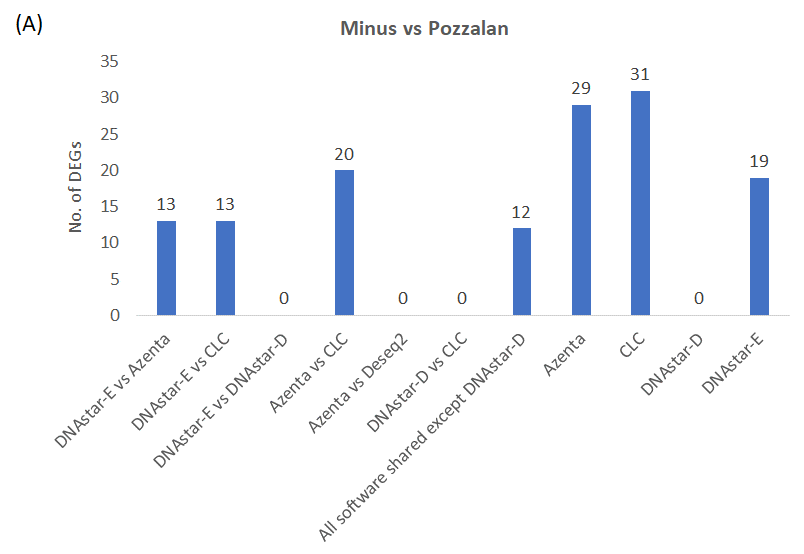

Supplementary Figure 2. Shared DEGs of mosquito (Aedes aegypti) among different software. (A) Minus vs Pozzalan treatment, (B) Minus vs Surface treatment and (C) Minus vs KCL treatment.

**Supplementary Table 1.** The raw reads obtained from RNA-seq of *E. coli* in this study.

| **Sample** | **Treatment** | **Raw reads** | **Raw data** | **Effective(%)** | **Error(%)** | **Q20(%)** | **Q30(%)** | **GC(%)** |
| --- | --- | --- | --- | --- | --- | --- | --- | --- |
| E1 | Minus | 22,158,658 | 3.32E+09 | 98.85 | 0.02 | 98.37 | 95.09 | 51.47 |
| E2 | Minus | 18,285,526 | 2.74E+09 | 99.12 | 0.02 | 98.29 | 94.84 | 51.48 |
| E3 | Minus | 17,394,852 | 2.61E+09 | 99.42 | 0.02 | 98.32 | 94.91 | 51.42 |
| E4 | Minus | 19,538,750 | 2.93E+09 | 99.49 | 0.03 | 97.98 | 94 | 51.45 |
| E5 | 1x KCL | 19,715,254 | 2.96E+09 | 99.51 | 0.02 | 98.22 | 94.66 | 51.55 |
| E6 | 1x KCL | 15,995,078 | 2.4E+09 | 99.44 | 0.02 | 98.39 | 95.07 | 51.39 |
| E7 | 1x KCL | 23,091,918 | 3.46E+09 | 99.48 | 0.02 | 98.26 | 94.82 | 51.39 |
| E8 | 1x KCL | 22,210,780 | 3.33E+09 | 99.42 | 0.02 | 98.37 | 95.05 | 51.56 |
| E9 | 2x KCL | 20,400,568 | 3.06E+09 | 99.51 | 0.02 | 98.34 | 94.98 | 51.38 |
| E10 | 2x KCL | 28,607,576 | 4.29E+09 | 99.53 | 0.02 | 98.33 | 94.95 | 51.43 |
| E11 | 2x KCL | 20,865,312 | 3.13E+09 | 99.49 | 0.03 | 97.35 | 92.77 | 51.51 |
| E12 | 2x KCL | 17,285,992 | 2.59E+09 | 99.23 | 0.03 | 97.59 | 93.34 | 51.47 |
| E13 | 0.5x KCL | 20,044,774 | 3.01E+09 | 99.2 | 0.03 | 97.66 | 93.35 | 51.34 |
| E14 | 0.5x KCL | 19,498,382 | 2.92E+09 | 99.37 | 0.03 | 97.6 | 93.32 | 51.35 |
| E15 | 0.5x KCL | 22,740,828 | 3.41E+09 | 99.33 | 0.03 | 97.57 | 93.27 | 51.5 |
| E16 | 0.5x KCL | 14,804,618 | 2.22E+09 | 99.41 | 0.03 | 97.65 | 93.43 | 51.5 |
| E17 | Surface | 19,210,532 | 2.88E+09 | 99.52 | 0.03 | 97.38 | 92.79 | 51.48 |
| E18 | Surface | 19,886,770 | 2.98E+09 | 99.41 | 0.03 | 97.62 | 93.26 | 51.46 |
| E19 | Surface | 30,246,862 | 4.54E+09 | 99.49 | 0.03 | 97.49 | 93.08 | 51.39 |
| E20 | Surface | 20,465,148 | 3.07E+09 | 99.54 | 0.03 | 97.25 | 92.51 | 51.42 |

**Supplementary Table 2**. The raw reads obtained from RNA-seq of Ae. aegypti

| **Sample ID** | **Treatment** | **# Reads** | **Yield (Mbases)** | **Mean Quality Score** | **% Bases >= 30** |
| --- | --- | --- | --- | --- | --- |
| JW3 | Minus | 52,034,316 | 15,610 | 35.67 | 92.41 |
| JW4 | Pozzalan | 49,854,297 | 14,956 | 35.66 | 92.39 |
| JW6 | Pozzalan | 53,999,693 | 16,200 | 35.89 | 93.60 |
| JW7 | KLC | 45,808,224 | 13,742 | 36.21 | 95.28 |
| JW8 | KCL | 52,845,902 | 15,854 | 35.92 | 93.71 |
| JW10 | Surface | 51,017,514 | 15,305 | 35.85 | 93.36 |
| JW12 | Surface | 53,874,823 | 16,162 | 35.87 | 93.46 |
| JW9 | KCL | 52,728,829 | 15,819 | 35.88 | 93.53 |
| JW1 | Minus | 48,513,726 | 14,554 | 35.67 | 92.39 |
| JW5 | Pozzalan | 59,842,094 | 17,953 | 35.88 | 93.58 |
| JW11 | Suface | 56,410,513 | 16,923 | 35.88 | 93.56 |
| JW2 | Minus | 50,817,699 | 15,245 | 35.63 | 92.24 |
